# 3-Hydroxypropanoic acid contributing to pollen abortion in ogura cytoplasmic male sterility of *Brassica napus*

**DOI:** 10.1186/s12870-026-09278-z

**Published:** 2026-06-18

**Authors:** Tanliu Wang, Jianghua Shi, Ying Fu, Huasheng Yu, Yaofeng Zhang, Xiyuan Ni

**Affiliations:** Key Laboratory of Digital Upland Crops of Zhejiang Province, Institute of Crop and Nuclear Technology Utilization, Zhejiang Academy of Agricultural Sciences, Hangzhou, China

**Keywords:** *Brassica napus* L., Cytoplasmic male sterility (CMS), Ogura-type CMS, Sterility mechanism, 3-hydroxypropionic acid (3-HP)

## Abstract

**Background:**

The Ogura-type male sterile cytoplasm is commonly used in *Brassicaceae* plants, but its sterility mechanism still requires further investigation.

**Results:**

The flower buds of Ogura CMS line ZJCMS1A and its maintainer line ZJCMS1B based on their length < 2 mm, 2 mm-3 mm, 3 mm-4 mm, grouped A2, A3, A4 and B2, B3, B4, respectively. Anthers were excised from flower buds of each group for transcriptome and metabolome sequencing. Differential comparison analysis, Venn analysis, Multi-omics Integration KEGG enrichment analysis, and pathway network analysis were performed on the data from different groups. The results showed that the abnormal propanoate metabolic pathway in ZJCMS1A was closely related to pollen abortion. The content of 3-hydroxypropionic acid (3-HP) in the propanoate metabolic pathway was higher in ZJCMS1A than in ZJCMS1B in the three comparisons A2 vs B2, A3 vs B3, and A4 vs B4. Furthermore, the content of 3-HP in ZJCMS2A flower buds was also higher than in ZJCMS2B. Quantification of 3-HP content revealed that the content in the stems, leaves, and flower buds of ZJCMS1A was higher than that in the corresponding tissues of ZJCMS1B. The 3-HP content in the flower buds of ZJCMS1A was 8.1% higher than that in ZJCMS1B. Treatment of rapeseed plants with 20 mM and 50 mM 3-HP showed that starch accumulation in pollen grains was affected.

**Conclusions:**

These results indicated that pollen abortion in Ogura-type CMS is closely related to abnormalities in the propanoate metabolic pathway, particularly the abnormal accumulation of 3-HP, which may participate in the pollen abortion process.

**Supplementary Information:**

The online version contains supplementary material available at https://doi.org/10.1186/s12870-026-09278-z.

## Introduction

Hybrid vigor refers to the phenomenon that the comprehensive traits of the offspring of two parents are better than those of the parents, Crops can increase yields through hybrid seeds, including corn, rice and rapeseed [36]. Male sterile plants can be used to produce hybrid seeds of crops and play an important role in utilizing hybrid vigor. Male sterility can be divided into cytoplasmic male sterility (CMS) and genic male sterility (GMS) according to the mode of inheritance. In CMS type the sterility gene is located in the mitochondrial genome, while in GMS the sterility gene is located in the nuclear genome [2]. In crop breeding, both CMS and GMS systems are widely used in the production of hybrid seeds. The CMS system, due to its ability to produce 100% male-sterile individuals at each generation, has significant advantages in ensuring seed purity [38].

There are many types of cytoplasmic male sterility in plants, but the mechanisms of action are currently summarized into four main models: the cytotoxicity model, the energy shortage model, the abnormal programmed cell death model and the reverse signal model. the cytotoxicity model, the energy shortage model, the abnormal cell programmed death model and the reverse signal model. The cytotoxic model believes that plant pollen abortion is due to the toxicity of CMS protein, which can directly kill pollen cells. The CMS protein URF13 of maize CMS-T type, ORF522 (CMS-PET1) in sunflower, ORF138 (CMS-Ogu) in radish, ORF288 (CMS-Hau) in cabbage, ORF79 (CMS-BT) and WA352 (CMS-WA) in rice have all been reported to be toxic to Escherichia coli cells [3, 5, 17, 22, 26, 28, 40]. However, there is currently a lack of direct verification evidence of this toxic effect in plant cells. The energy shortage model indicates that CMS protein causes mitochondrial dysfunction, which results in energy deficiency during reproductive development and pollen abortion [21]. CMS protein may compete with the components of the mitochondrial electron transport chain (mtETC) or F-type proton transport ATPase (F_1_F_o_-ATPase) complex for some binding sites, affecting their normal formation and causing their dysfunction, thus causing male sterility [2]. ORFH 79 in rice CMS-HL was confirmed to interact with the subunit P61 of complex III in the mitochondrial electron transport chain, thereby reducing the activity of complex III and leading to a decrease in ATP [39]. ORF522 in sunflower CMS-PET1 may reduce the activity of F_1_F_o_-ATPase and cause sterility [32]. In addition, many CMS proteins have mitochondrial transmembrane structures, such as URF13 in maize CMS-T, ORF138 in radish CMS-Ogu, and ORF79 in rice CMS-BT, the transmembrane structures of these proteins may integrate these proteins into the inner membrane of mitochondria and affect ATP synthesis [16, 19, 31]. The abnormal programmed cell death (PCD) model mainly refers to the fact that premature or delayed PCD of the tapetum will lead to male sterility [18]. Because the tapetum not only provides energy for the formation of male gametes, but also is the material basis for the development of male gametes, the timely PCD of the tapetum to cause anther dehiscence also constitutes the structural basis for the function of male gametes. ORF522 in sunflower CMS-PET1 causes premature PCD of the tapetum, thus causing male sterility [1], and WA352 in CMS-WA in rice also causes premature PCD of the tapetum, leading to the occurrence of male sterility [26]. The retrograde-regulated model was first discovered in rice. The sterility gene of rice CMS-CW is *orf307*, and its restorer gene *Rf17* and restorer allele *rf17* cannot affect the transcription of *orf307*. However, there is a reverse signal *RMS* in the cytoplasm of CMS-CW, which can upregulate the expression of *rf17* without upregulating *Rf17*, thereby leading to male sterility [9].

Cruciferous crops are of great value in agricultural production, and cytoplasmic male sterility systems play an important role in the heterosis utilization of these crops. At least 16 types of CMS systems have been discovered in cruciferous crops including Ogura CMS, Polima CMS, Kosena CMS and Moricandia arvensis CMS, among others [29, 30], [41]. The advantage of the Ogura CMS system is that its pollen abortion is thorough, making it an ideal choice for hybrid seed production, so researchers have conducted in-depth research and applied it extensively. The Ogura CMS system has been applied to a variety of economic crops, including *Raphanus sativus*, *Brassica napus*, *Brassica oleracea*, *Brassica rapa*, and *Brassica juncea*, and related research has been conducted [12, 24, 42, 44]. In plant research, both overexpression and knockout of the *orf138* gene have demonstrated its crucial role in Ogura CMS, the *orf138* was found to be generated by mitochondrial genome rearrangement through comparative analysis of the mitochondrial genome [10, 11, 35, 43]. Studies have shown that ORF138 has toxic effects on cells. Through a large number of omics analyses, especially transcriptome analyses, of Ogura CMS lines from different cruciferous crop backgrounds, it was found that DEGs involved a wide variety of KEGG pathways, including energy metabolism, pollen formation, redox reaction, apoptosis, etc. [4, 13, 43], 47. Transcriptome sequencing of anthers before and after abortion of Ogura CMS in *Brassica napus* revealed that ORF138 may lead to abnormal lipid metabolism and causing cytoplasmic male sterility in Ogura CMS [10]. However, current studies on pollen abortion process in Ogura cytoplasmic male sterile lines suffer from several challenges. The sample collection is not precise enough, most experiments focusing on anthers include petals, sepals and pistils. Furthermore, distinguishing the stages of pollen abortion is also difficult. These problems result in inaccurate experimental data, hindering in-depth analysis of the mechanisms of Ogura cytoplasmic male sterility.

Here, through microscopic observation, we found that pollen grains in ZJCMS1A were slightly wrinkled when the flower bud length was 2 mm, and completely shriveled and wrinkled when the flower bud length was 3 mm. Based on flower bud length, we divided the buds into early abortion stage (< 2 mm), middle abortion stage (2 mm-3 mm), and late abortion stage (3 mm-4 mm). Anthers from each group were extracted for transcriptome and metabolome sequencing. Analysis revealed 3308 differentially expressed genes between the anthers of ZJCMS1A (A2) and ZJCMS1B (B2) in the early abortion stage (< 2 mm). The results of pollen grain microscopic observation and transcriptome data analysis indicate that pollen abortion in the Ogura CMS line ZJCMS1A likely begins in the early abortion stage (< 2 mm). At the 2–3 mm flower bud length stage, 24,180 differentially expressed genes were found between the anthers of ZJCMS1A (A3) and ZJCMS1B (B3), indicating pollen grains complete abortion. Metabolomics data analysis revealed similar levels of metabolite changes in the early abortion stage and the middle abortion stage, A2_vs_B2 has 738 different metabolites and A3_vs_B3 has 796 different metabolites, suggesting that during the Ogura-type CMS process, metabolic changes are already quite extensive. Venn analysis was performed on the differentially expressed genes and metabolites between the anthers of ZJCMS1A and ZJCMS1B at different developmental stages to identify pollen abortion-related genes and metabolites. Multi-omics integration KEGG analysis identified 15 pollen abortion-related KEGG maps. Analysis revealed 154 differentially expressed genes and 58 differentially expressed metabolites specifically present between A2 and B2, belonging to the aforementioned 15 KEGG maps. Enrichment analysis showed they were mainly related to the propanoate metabolic pathway and the fatty acid biosynthesis pathway, the KGML analysis revealed a correlation between these two pathways. Pathway diagram analysis revealed that the expression level of 3-hydroxypropionyl-CoA in the propanoate metabolic pathway at different anther developmental stages differs between ZJCMS1A and ZJCMS1B. Moreover, the content of 3-hydroxypropionic acid in the propanoate metabolic pathway was higher in ZJCMS1A than in ZJCMS1B, and this difference was only observed in the anthers of ZJCMS1A and ZJCMS1B at the same developmental stage. Furthermore, we found that the 3-hydroxypropionic acid content in mixed flower buds of Ogura-type cytoplasmic male sterile line ZJCM2A was also higher than that in ZJCMS2B. Chemical analysis of 3-hydroxypropionic acid content in ZJCMS1A and ZJCMS1B flower buds showed that the content in ZJCMS1A flower buds was 8.1% higher than that in ZJCMS1B. Furthermore, the content of 3-hydroxypropionic acid in the stem and leaves of ZJCMS1A was also higher than that of ZJCMS1B. Experiments have shown that continuous spraying of rapeseed plants with 20 mM and 50 mM 3-hydroxypropionic acid during the initial flowering stage affects starch accumulation in pollen grains.

## Results

### Relationship between the pollen abortion period and flower bud size of ZJCMS1A

**Fig. 1:**
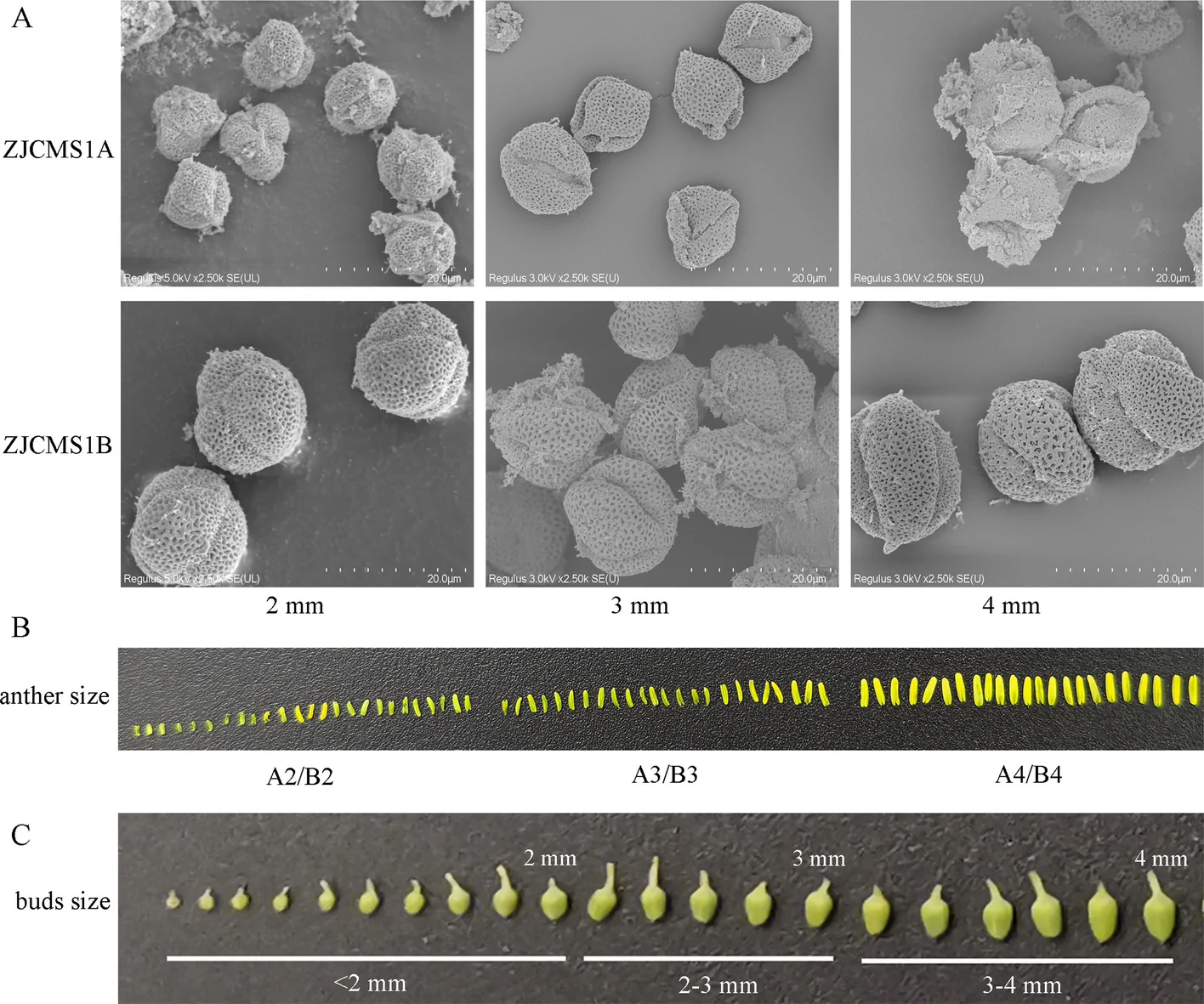
Classification of flower buds and microscopic observation of pollen grains. **A** Observation of pollen grains in different length flower buds of ZJCMS1A and ZJCMS1B using a scanning electron microscope. Bar = 20 μm. **B** Classification of anthers in different length flower buds of ZJCMS1A/ZJCMS1B. A2 represents anthers in flower buds of ZJCMS1A with a length less than 2 mm. A3 represents anthers in flower buds of ZJCMS1A with a length between 2 and 3 mm. A4 represents anthers in flower buds of ZJCMS1A with a length between 3 and 4 mm. B2 represents anthers in flower buds of ZJCMS1B with a length less than 2 mm. B3 represents anthers in flower buds of ZJCMS1B with a length between 2 and 3 mm. B4 represents anthers in flower buds of ZJCMS1B with a length between 3 and 4 mm. Transcriptomic and metabolomic sequencing were performed on samples A2, A3, A4, B2, B3, and B4. **C** The flower buds of ZJCMS1A and ZJCMS1B were classified according to their length

ZJCMS1A is a male sterile line with Ogura-type cytoplasm in *Brassica napus*, and ZJCMS1B is the corresponding maintainer line *Zheyou51*. The pollen grain morphologies in flower buds of different sizes were observed in ZJCMS1A and ZJCMS1B, respectively. It was found that when the flower bud size was 2 mm, the pollen grains in ZJCMS1A and ZJCMS1B were structurally intact, but the pollen grains in ZJCMS1A were slightly deformed. In the 3 mm flower buds, the pollen grains in ZJCMS1A had become shriveled and wrinkled. In the 4 mm flower buds, the pollen grains in ZJCMS1A had become damaged, losing the integrity and plump structure of normal pollen in ZJCMS1B (Fig. 1A). It was confirmed that when the flower bud length reached 4 mm, the pollen grains in ZJCMS1A were completely aborted.

The flower buds of ZJCMS1A and ZJCMS1B were divided into three groups according to their sizes of less than 2 mm, 2–3 mm, and 3–4 mm, respectively. The anthers were peeled off according to the specified flower bud size of each group, and divided into the early abortion stage A2 (ZJCMS1A-2, < 2 mm), middle abortion stage A3 (ZJCMS1A-3, 2–3 mm), and late abortion stage A4 (ZJCMS1A-4, 3–4 mm) were obtained, as well as the corresponding control group B2 (ZJCMS1B-2, < 2 mm), B3 (ZJCMS1B-3, 2–3 mm), and B4 (ZJCMS1B-4, 3–4 mm) (Fig. 1B; Fig. 1C). The differences between the anthers of ZJCMS1A and ZJCMS1B at the same stage were primarily at the pollen in microscopic level.

### Metabolites are more abundant than genes in the early abortion stage of ZJCMS1A

**Fig. 2:**
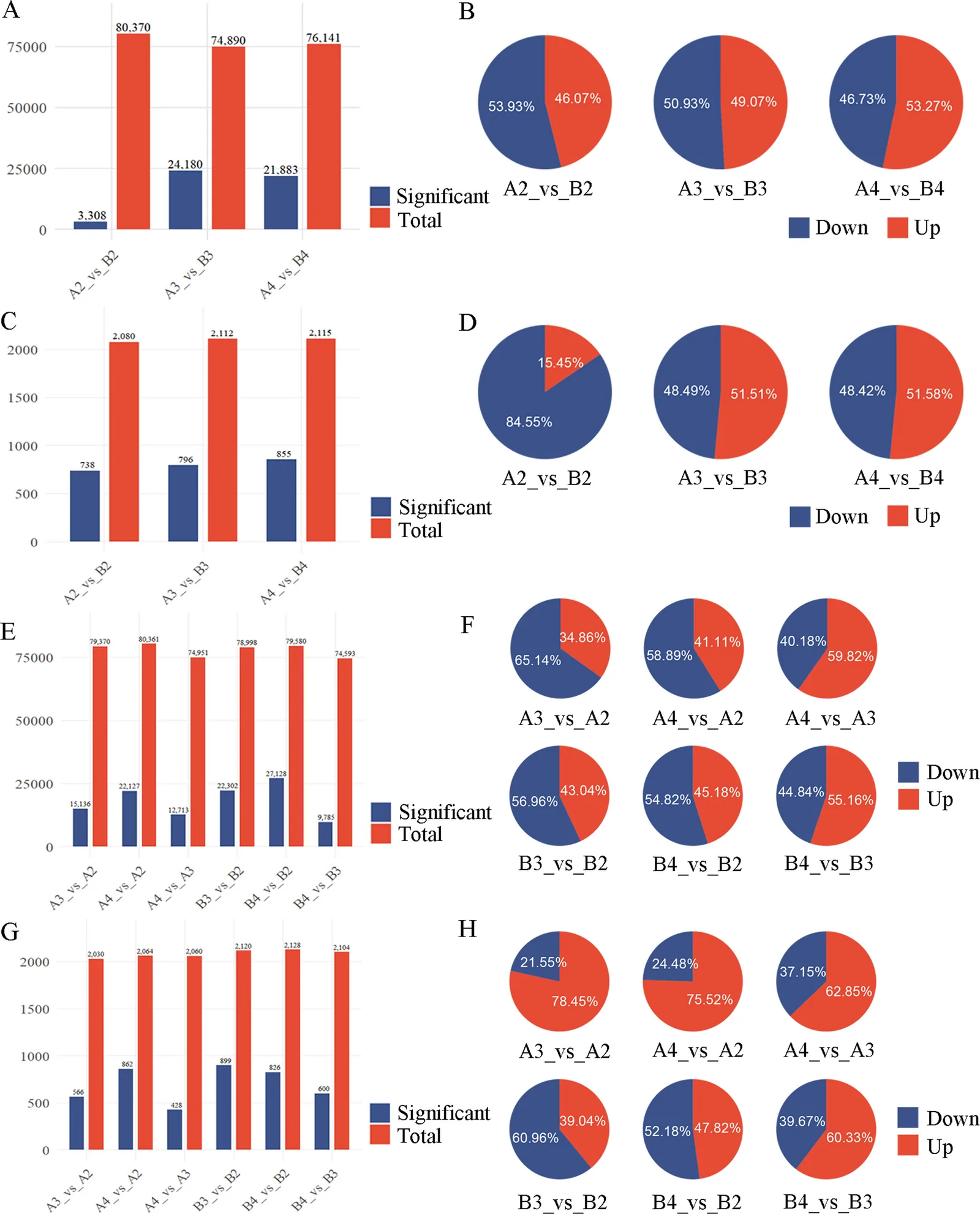
Number and proportion of differentially expressed genes or differentially metabolites between different samples. **A** Number of differentially expressed genes between different samples. **B** Proportion of upregulated and downregulated genes between different samples. **C** Number of differentially metabolites between different samples. **D** Proportion of upregulated and downregulated metabolites between different samples. **E** Number of differentially expressed genes between different samples. **F** Proportion of upregulated and downregulated genes between different samples. **G** Number of differentially metabolites between different samples. **H** Proportion of upregulated and downregulated metabolites between different samples

Transcriptomic and metabolomic sequencing were performed on the anthers of ZJCMS1A and ZJCMS1B at different developmental stages. Comparison of gene expression levels during the same developmental stages revealed significant changes in 3,308 genes between A2 and B2. This suggests that when the flower bud is less than 2 mm, the expression levels of a small number of genes in ZJCMS1A anthers change under the influence of the Ogura sterile cytoplasm, indicating an early stage of abortion. When the flower bud size is 2–3 mm, the number of differentially expressed genes between ZJCMS1A and ZJCMS1B anthers reaches 24,180, indicating an active stage of abortion. When the flower bud size is 3–4 mm, abortion has reached a late stage, and the number of differentially expressed genes decreases to 21,883 (Fig. 2A). As development progressed, the number of genes upregulated in ZJCMS1A anthers gradually increased. (Fig. 2B). The patterns of differential gene changes at different developmental stages are consistent with the patterns of changes in pollen appearance observed through microscopy.

Comparison of metabolite differences during the same developmental stages revealed changes in 738, 796, and 855 metabolites in A2_vs_B2, A3_vs_B3, and A4_vs_B4, respectively (Fig. 2C). This suggests that during the early abortion stage, when flower buds are less than 2 mm, metabolite changes in ZJCMS1A anthers have reached the level seen during the active abortion period. However, the number of genes with differential expression levels during the early abortion stage was much lower than during the active abortion period, indicating that ORF138-induced changes in metabolism are greater than those in gene expression during the early abortion stage of ZJCMS1A caused by Ogura sterile cytoplasm. Comparison of the up- and down-regulated proportions of differentially expressed metabolites revealed that 84.55% of metabolites were down-regulated in A2_vs_B2, while 48.49% and 48.42% were down-regulated in A3_vs_B3 and A4_vs_B4, respectively (Fig. 2D). This suggests that during the early stages of abortion, multiple metabolic pathways may be impaired in ZJCMS1A anthers, leading to abnormalities in the synthesis and metabolism of multiple metabolites, resulting in the down-regulation of numerous metabolites in A2.

Gene expression levels and metabolite changes were compared between ZJCMS1A and ZJCMS1B at different developmental stages. There were 15,136 differentially expressed genes in A3_vs_A2 and 22,302 in B3_vs_B2, with fewer gene changes in ZJCMS1A than in ZJCMS1B. There were 566 and 899 differentially metabolites in A3_vs_A2 and B3_vs_B2, respectively, with trends consistent with those for differentially expressed genes (Fig. 2E and G). The low number of differentially expressed genes and metabolites suggests that the developmental defects caused by the Ogura sterile cytoplasm in ZJCMS1A anthers during A2-A3 development leads to the dysfunction of certain genes and the abnormal synthesis and metabolism of specific metabolites. Analysis revealed that 65.14% of genes were downregulated in the A3_vs_A2 comparison, compared to 56.96% in the B3_vs_B2 comparison. 78.45% of metabolites were upregulated in the A3_vs_A2 comparison, compared to only 39.04% in the B3_vs_B2 comparison (Fig. 2F). These abnormally altered genes and metabolites are very likely involved in the pollen abortion process of ZJCMS1A, indicates the A2-A3 phase is the occurrence stage where pollen abortion of ZJCMS1A. A4_vs_A3 showed 12,713 differentially expressed genes and 428 metabolites, all of which were reduced compared to A3_vs_A2 comparisons. This suggests that ZJCMS1A anther development is more active during the A2-A3 phase than during the A3-A4 phase, with more changes in genes and metabolites (Fig. 2E and G). ZJCMS1B also exhibited similar characteristics (Fig. 2E and G). Analysis revealed that the up- and down-regulation level between differentially expressed genes and metabolites in A4_vs_A3 and B4_vs_B3 phases were essentially identical, the changes tended to be stable (Fig. 2H), indicating that A3-A4 is the final phase of pollen abortion in ZJCMS1A. These results are consistent with the microscopic observations.

### Functional analysis of differentially expressed genes and metabolites during pollen abortion in ZJCMS1A

**Fig. 3:**
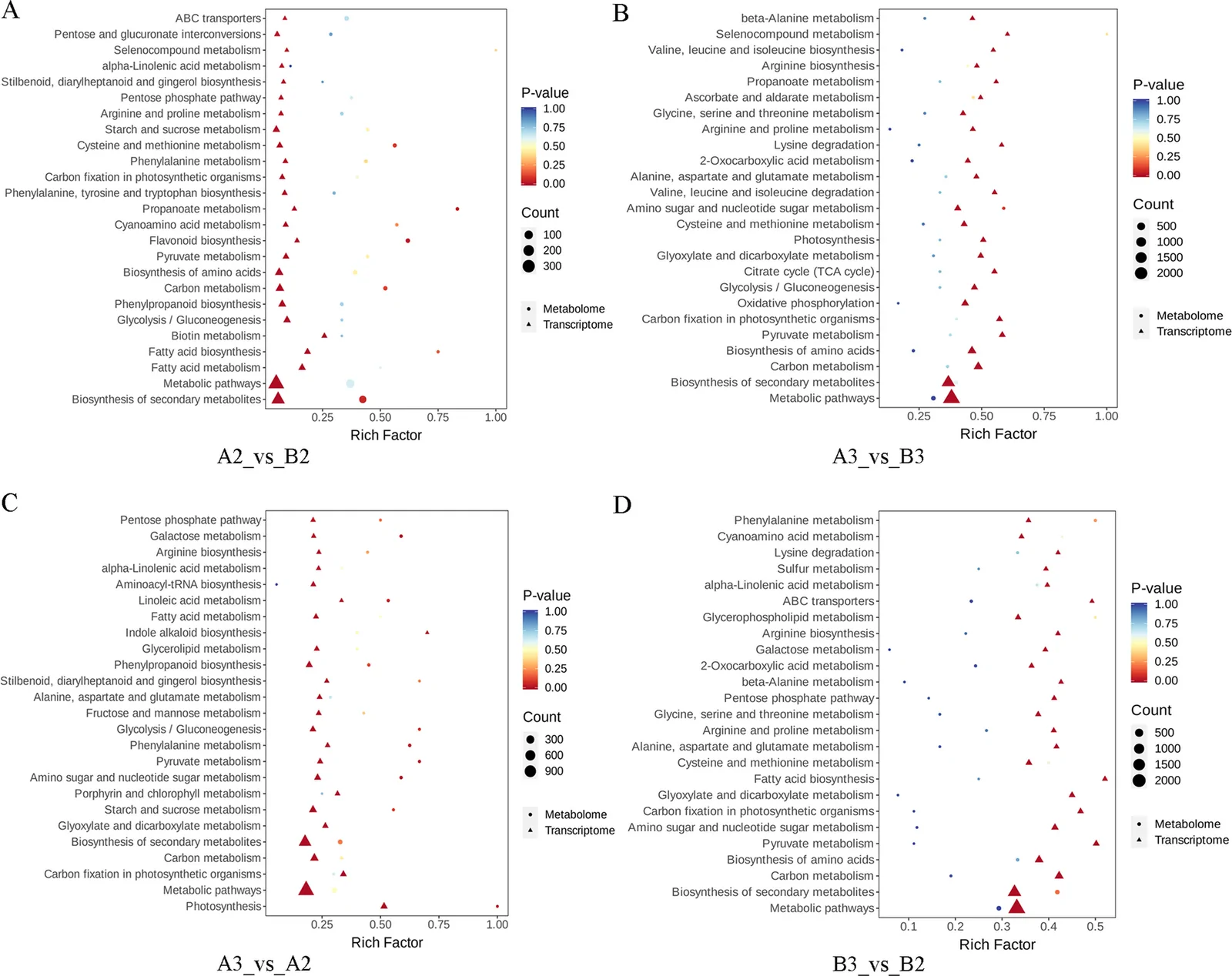
Multi-omics Integration KEGG Analysis. **A** Multi-omics integrated KEGG analysis between samples A2 and B2. **B** Multi-omics integrated KEGG analysis between samples A3 and B3. **C** Multi-omics integrated KEGG analysis between samples A3 and A2. **D** Multi-omics integrated KEGG analysis between samples B3 and B2

Microscopic observation and comparative analysis of differentially expressed genes and metabolites identified A2 as the early stage of pollen abortion in ZJCMS1A, and A3 was the active stage of abortion. KEGG enrichment analysis of the differentially expressed genes and metabolites between A2 and B2 revealed that the main enriched pathways are propanoate metabolism (Fig. 3A), which primarily metabolizes propionic acid produced by the degradation of branched-chain amino acids and is a hub connecting sugar metabolism, amino acid metabolism, and energy metabolism. As an organic acid, propionic acid may participate in cellular pH regulation and oxidative stress buffering in plants. In addition, flavonoid biosynthesis and fatty acid biosynthesis were also enriched (Fig. 3A). The former is primarily related to plant antioxidant activity, while the latter is a pathway for the synthesis of fatty acids. Fatty acids are important components of phospholipids in cell membranes and are crucial for maintaining cell membrane structure and fluidity. These biological processes are closely related to pollen abortion.

KEGG enrichment analysis of A3_vs_B3 revealed that the propanoate metabolic pathway remained enriched (Fig. 3B). Amino sugar and nucleotide sugar metabolism are enriched pathways, providing sugar donors for the cell, forming precursors for the synthesis of polysaccharides such as cellulose and sporopollenin, and participating in pollen wall synthesis and energy supply. Pyruvate metabolism and lysine degradation are related to energy supply. During the development of ZJCMS1A from A2 to A3, the differentially expressed genes and metabolites were mainly involved in photosynthesis, which related to energy production, pyruvate metabolism related to energy supply, and linoleic acid metabolism related to cell membrane phospholipid synthesis (Fig. 3C).

The development of ZJCMS1B anther from B2 to B3 is a normal fertile anther development process. The metabolites and genes that changed between the two stages were all involved in biological processes necessary for anthers development. The enriched fatty acid biosynthesis (Fig. 3D), a core metabolic pathway for energy storage and membrane construction in plants, was also enriched in A2_vs_B2. Pyruvate, a precursor for cellular energy supply and biomacromolecule synthesis, is enriched in both B3_vs_B2 and A3_vs_A2. Transmembrane transport is crucial for cellular acquisition of materials, removal of metabolic waste, and defense against toxins. ABC transporters are crucial transmembrane transport proteins, and the ABC transporter pathway is enriched in B3_vs_B2. Furthermore, pathways related to glycerophospholipid metabolism, a core component of biomembranes, and carbon fixation in photosynthetic organisms, which are related to plant carbon fixation, are also enriched in B3_vs_B2.

### Functional analysis of specific differentially expressed genes and metabolites

**Fig. 4:**
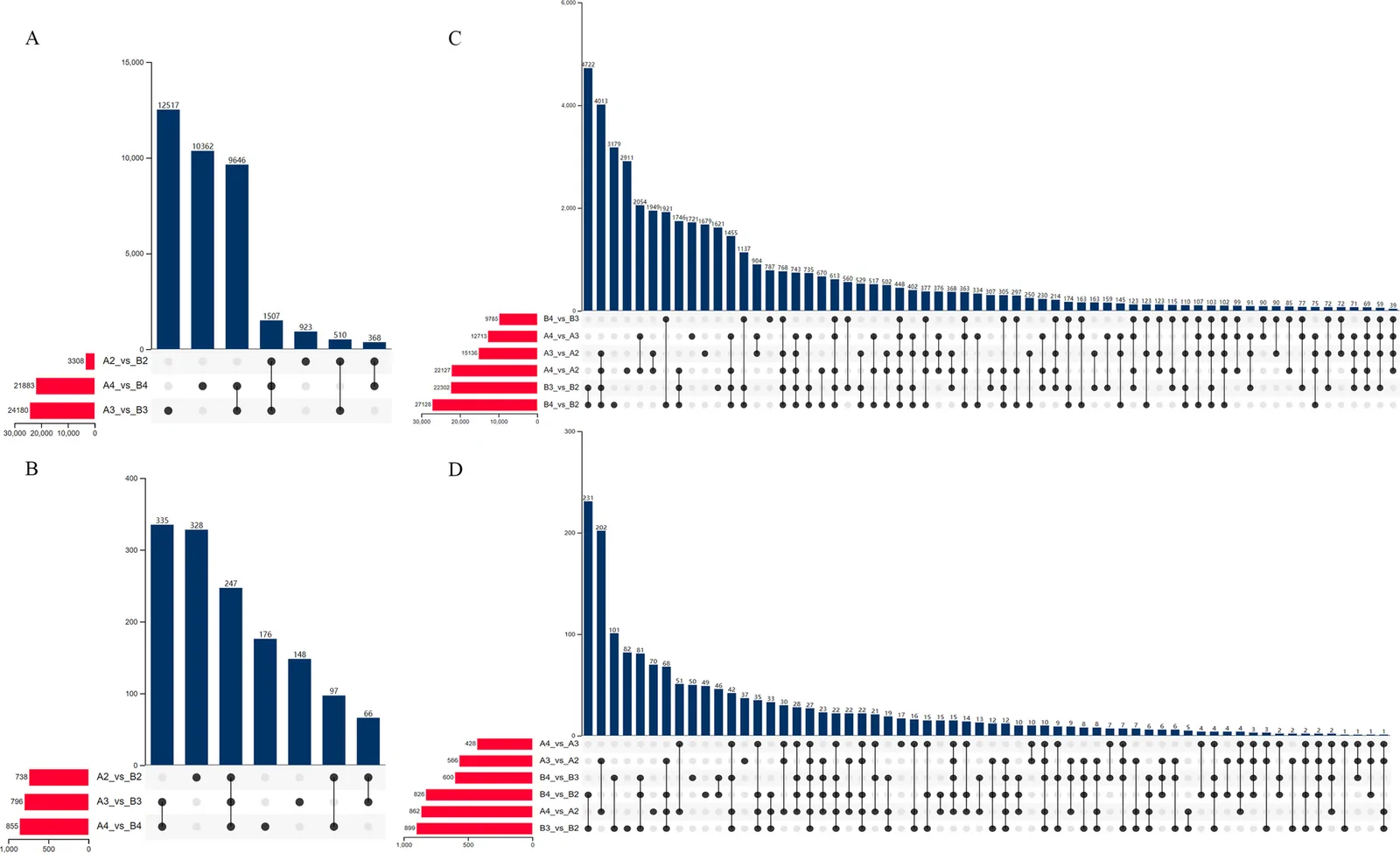
Venn diagram analysis of differentially expressed genes or differentially metabolites between different comparison groups. **A** Venn analysis of differentially expressed genes among the 3 comparison groups. **B** Venn analysis of differentially metabolites among the 3 comparison groups. **C** Venn analysis of differentially expressed genes among the 6 comparison groups. **D** Venn analysis of differentially metabolites among the 6 comparison groups

During pollen abortion in ZJCMS1A, numerous genes and metabolites change. To identify the functions of genes and metabolites directly associated with pollen abortion, we performed Venn analysis on differentially expressed genes and differential metabolites at stages A2_vs_ B2, A3_vs_B3, and A4_vs_B4, respectively. We found 923 differentially expressed genes and 328 differential metabolites specific to A2_vs_B2 (Fig. 4A and B). Analysis of the metabolic pathways involved in these 923 genes and 328 metabolites revealed enrichment in propanoate metabolic pathways and pyruvate metabolic pathways, which are associated with energy supply, as well as the glycolipid metabolic pathway, which is associated with membrane structure (S Fig. 1. A). A2 is the early stage of pollen abortion, this suggests that pollen abortion in ZJCMS1A is closely related to energy and membrane structure. Both 1,507 differentially expressed genes and 247 differential metabolites were found in all A2_vs_B2, A3_vs_B3, and A4_vs_B4 (Fig. 4A and B). KEGG enrichment analysis revealed that the stilbenoid, diarylheptanoid and gingerol biosynthesis pathways and the flavonoid biosynthesis pathway were enriched, which are related to antioxidant (S Fig. 1. B), suggesting that ROS burst may occur during pollen abortion in ZJCMS1A.

Compared with ZJCMS1B, 376 differentially expressed genes and 30 differential metabolites were specifically existed during the A2, A3, and A4 developmental stages in ZJCMS1A (Fig. 4C and D). These genes and metabolites were primarily involved in the antioxidant-related stilbenoid, diarylheptanoid, and gingerol biosynthesis pathways (S Fig. 1. C). Similarly, both 1137 differentially expressed genes and 81 differential metabolites were specifically existed during the B2, B3, and B4 developmental stages in ZJCMS1B (Fig. 4C and D). These genes and metabolites were enriched in the ubiquinone and other terpenoid-quinone biosynthesis pathways, which are related to electron transport and antioxidant activity in biological membranes, the ABC transporters pathway involved in transmembrane transport, and the flavonoid biosynthesis pathway related to antioxidant activity (S Fig. 1. D).

Analysis of differentially expressed genes and differential metabolites specifically in ZJCMS1A and ZJCMS1B revealed that ROS burst may occur during the abortion process of ZJCMS1A, while the comprehensive regulation of genes and metabolites related to the electron transport chain, antioxidants, and transmembrane transport in ZJCMS1B maintains anther development and pollen fertility.

### Analysis of important KEGG pathways related to pollen abortion in ZJCMS1A

**Fig. 5:**
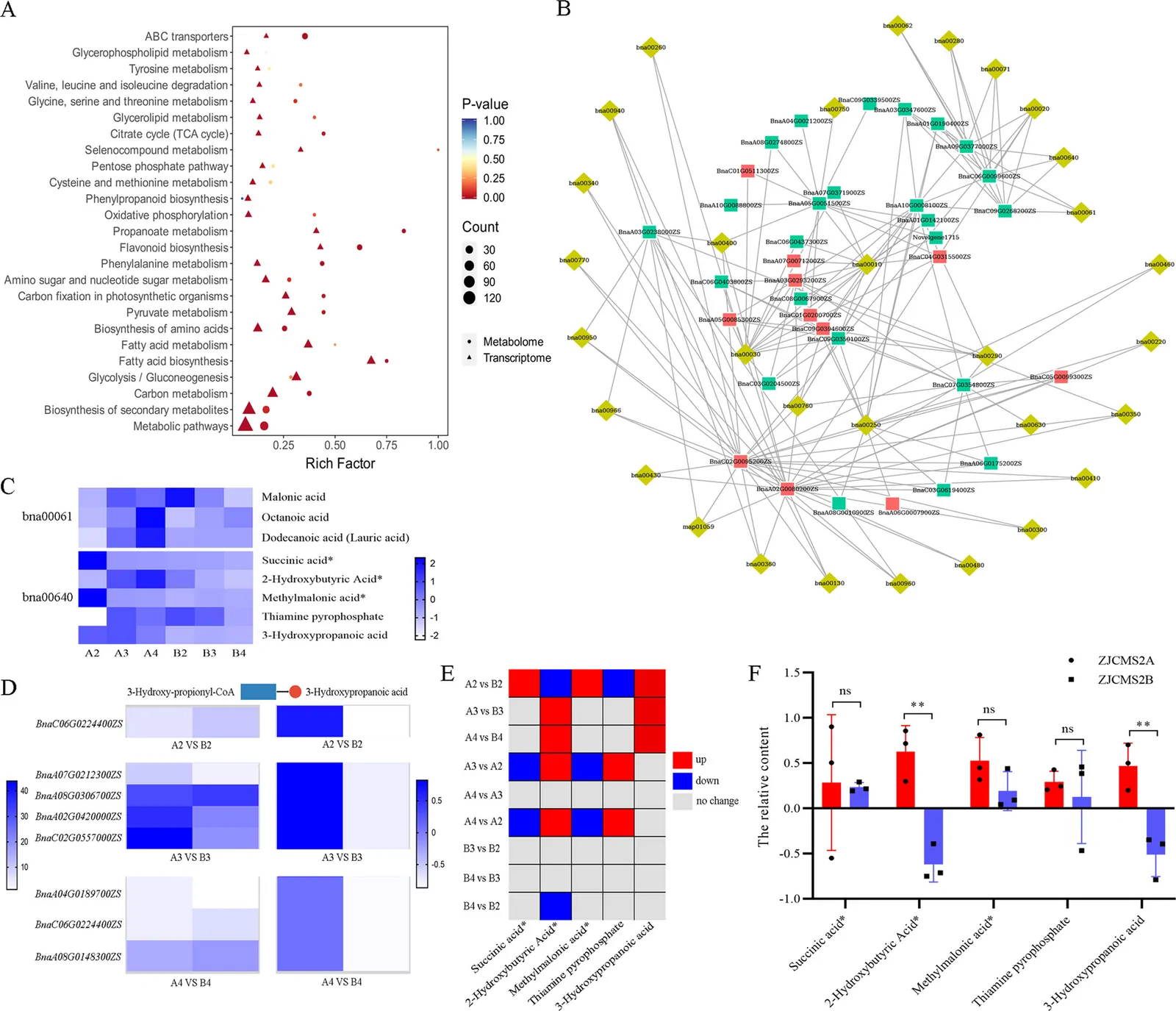
Pathway analysis and substance content analysis. **A** Multi-omics Integration KEGG Analysis of differentially expressed genes and differential metabolites involved in 14 key KEGG maps in the A2_vs_B2 comparison group. **B** KEGG Markup Language (KGML) analysis of differentially expressed genes and differential metabolites involved in 14 key KEGG maps in the A2_vs_B2 comparison group. **C** Metabolite content in different samples from metabolomics sequencing results. **D** Differentially expressed genes between different samples from transcriptomics sequencing results. **E** Differential metabolites between different samples from metabolomics sequencing results. **F** Metabolite content in the metabolomics sequencing results of ZJCMS2A and ZJCMS2B flower buds. Asterisks indicate significant differences, and ns indicates no significant difference. The significance of differences was compared using Student's t-test (Two-tailed)

KEGG enrichment analysis of differentially expressed genes and differential metabolites in the nine groups of comparisons between ZJCMS1A and ZJCMS1B revealed that 15 KEGG pathways were mainly involved in the pollen abortion process of ZJCMS1A (S Table 1). The A2 is the early stage of pollen abortion in ZJCMS1A, during which the ORF138 protein begins to function. There were 3,308 differentially expressed genes and 738 differential metabolites in A2_vs_B2, of which 163 genes and 58 metabolites were involved in 15 key KEGG pathways. Enrichment analysis of the functions of these 154 genes (without 9 novel genes) and 58 metabolites revealed that they were primarily enriched in the propanoate metabolic pathway, fatty acid biosynthesis pathway, and flavonoid biosynthesis pathway (Fig. 5A; S Table 2; S Table 3). Propanoate metabolic is associated with energy supply and biosynthesis (such as fatty acid synthesis) and can cause metabolic acidosis in humans. Fatty acid biosynthesis is associated with cell membrane structure and the sporopollenin synthesis in the pollen wall. Flavonoid biosynthesis is involved in ROS scavenging.

Pathway network analysis (KGML) of 163 differentially expressed genes and 58 differential metabolites involved in 15 key KEGG pathways in A2_vs_B2 revealed the correlation between bna00640 (Propanoate metabolism) and bna00061 (Fatty acid biosynthesis) (Fig. 5B). This suggests that the propanoate metabolic pathway and fatty acid biosynthesis pathway may play an important role in the pollen abortion process of ZJCMS1A. Analysis of the differential metabolites detected in the propanoate metabolic pathway and the fatty acid biosynthesis pathway, we revealed that only 3-hydroxypropanoic acid had high levels in A2, A3, and A4, and low levels in B2, B3, and B4 (Fig. 5C), indicating that 3-hydroxypropanoic acid in ZJCMS1A is likely to be abnormally metabolized, leading to accumulation and increase in its content. The KEGG map of the propanoate metabolic pathway revealed that 3-Hydroxy-propionyl-CoA is directly involved in the production of 3-hydroxypropanoic acid. Transcriptome data and qRT-PCR analysis revealed differences in 3-Hydroxy-propionyl-CoA expression between A2 and B2, A3 and B3, and A4 and B4 (Fig. 5D; S Fig. 2). The elevated 3-hydroxypropanoic acid content in ZJCMS1A is likely related to the abnormality of 3-Hydroxy-propionyl-CoA. Analysis of 3-Hydroxypropanoic acid content changes at different developmental stages in ZJCMS1A and ZJCMS1B revealed that differences exist only between A2_vs_B2, A3_vs_B3, and A4_vs_B4 (Fig. 5E), suggesting that 3-Hydroxypropanoic acid content is specifically elevated in ZJCMS1A. Furtherly, we conducted metabolomic analysis on mixed flower buds of another Ogura-type CMS rapeseed line (Brassica napus L.), ZJCMS2A, and its corresponding maintainer line, ZJCMS2B. We found that the content of 3-hydroxypropanoic acid in the flower buds of ZJCMS2A was also higher than that of ZJCMS2B (Fig. 5F; S Table 4). Analysis of key KEGG pathways associated with pollen abortion in ZJCMS1A and confirmation in ZJCMS2A, we finally revealed that the specific increase in 3-hydroxypropanoic acid levels is likely closely related to the pollen abortion of Brassica napus caused by Ogura-type sterile cytoplasm.

## Abnormal accumulation of 3-hydroxypropionic acid affects pollen fertility

**Fig. 6:**
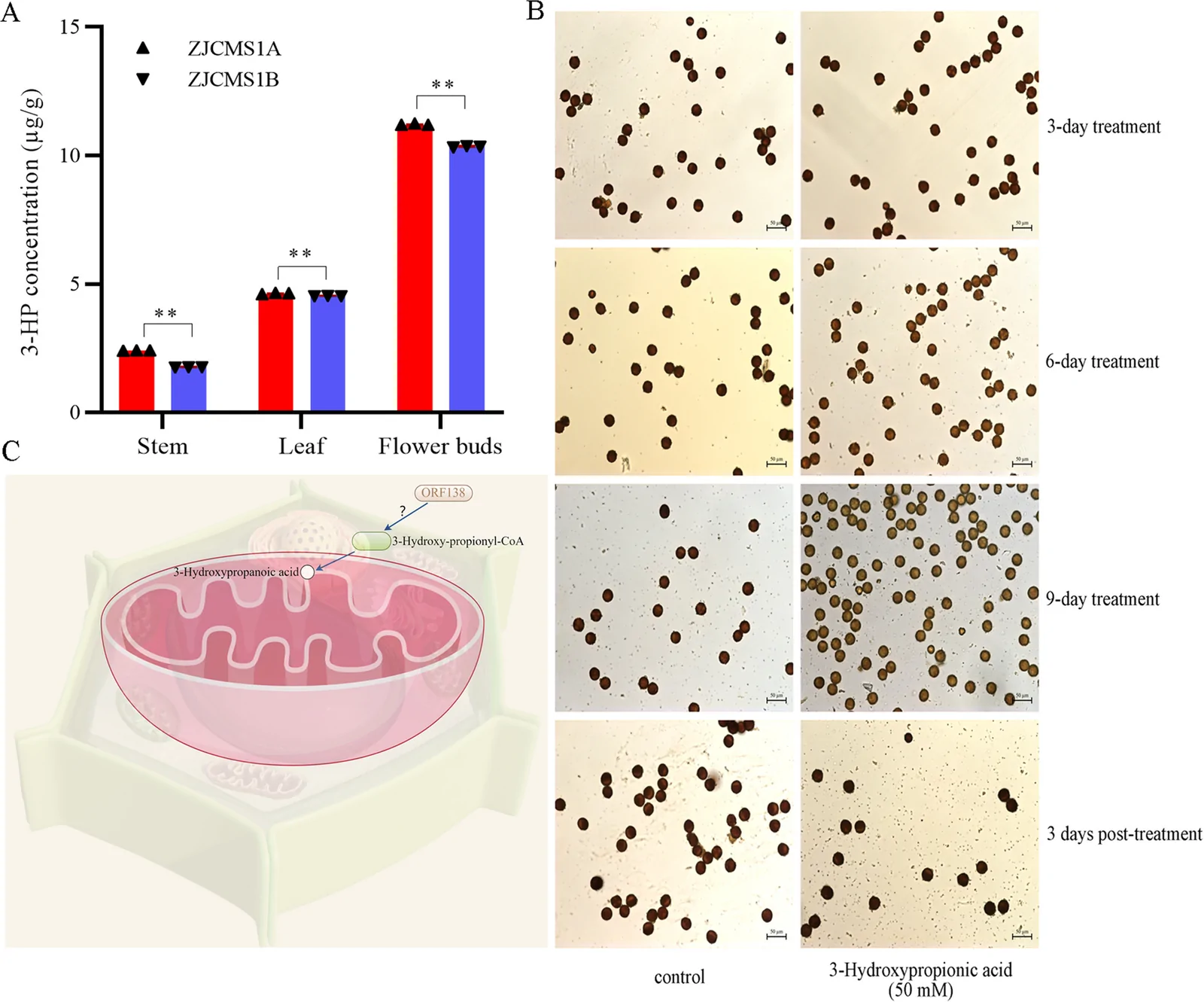
Determination of 3-hydroxypropionic acid content and microscopic observation of pollen grains. **A** Results of 3-hydroxypropionic acid content determination in different tissues of ZJCMS1A and ZJCMS1B. Asterisks indicate significant differences. The significance of differences was compared using Student's t-test (Two-tailed). **B** Rapeseed plants were sprayed with 50 mM 3-hydroxypropionic acid. Pollen grains were observed using iodine-potassium iodide staining on days 3, 6, and 9 after treatment, as well as on 3 days post-treatment. Darker color indicates higher starch content and better pollen viability. Lighter black indicates low starch content and abnormal pollen development. Darker color indicates higher starch content and better pollen grain viability. Lighter color indicates lower starch content and abnormal pollen grain development. **C** Schematic diagram of the mechanism of action of 3-hydroxypropionic acid

The 3-hydroxypropionic acid content was measured in different tissues of ZJCMS1A and ZJCMS1B. It was found that the content was higher in flower buds than in stems and leaves in both varieties. Furthermore, the 3-hydroxypropionic acid content in the stem, leaf, and flower buds of ZJCMS1A was significantly higher than that of ZJCMS1B. The 3-hydroxypropionic acid content in the flower buds of ZJCMS1A was 11.2 μg/g, which was an 8.1% increase compared to ZJCMS1B (Fig. 6A; S Table 5). Treating rapeseed plants in the early flowering stage with 50 mM 3-hydroxypropionic acid and observing pollen staining revealed that after 3 days of consecutive spraying, pollen grains in both the 50 mM 3-hydroxypropionic acid group and the control group were black, indicating normal starch accumulation. After 6 days of consecutive spraying, compared to the control group, the pollen grains of the rapeseed plants sprayed with 50 mM 3-hydroxypropionic acid were slightly yellow, indicating lower starch accumulation than the control group. After 9 days of consecutive spraying, the pollen grains in the sprayed group were significantly yellow, indicating insufficient starch accumulation. Three days after treatment was stopped, the pollen grain staining was consistent with the control group, indicating normal starch accumulation (Fig. 6B). In the 20 mM treatment group, starch accumulation in the pollen grains was also observed to be affected after 7 days of treatment (S Fig. 3). These results suggest that alterations in the content of 3-hydroxypropionic acid can impact starch accumulation in rapeseed pollen grains, thereby affecting rapeseed pollen fertility. Between the same developmental stages of ZJCMS1A and ZJCMS1B, A2_vs_B2/A3_vs_B3/A4_vs_B4, the expression levels of 3-hydroxypropionyl-CoA-related regulatory genes exhibit differences (S Fig. 2; S Table 6). The above results indicate that the expression of ORF138 in ZJCMS1A may affect the levels of 3-hydroxypropionyl-CoA and further influence the content of 3-hydroxypropanoic acid (Fig. 6C). An increase in 3-hydroxypropanoic acid content may exacerbate mitochondrial damage in pollen grains, which closely associated with pollen abortion.

## Discussion

Cytoplasmic male sterility is a common phenomenon in plants, but the mechanism by which CMS genes cause male sterility has long puzzled researchers. One of the currently proposed hypothetical models for CMS sterility is the cytotoxicity model, which proposes that CMS proteins are toxic and can directly kill cells [2]. ORF522 in the sunflower CMS-PET1, ORF288 of Hau CMS, and ORF346 of Nsa CMS are toxic to Escherichia coli [17, 28, 33]. The toxicity of WA352 from rice CMS-WA to Escherichia coli depends on its transmembrane structure, and recent studies have shown that ubiquitination of the transmembrane region of WA352 leads to loss of function [2, 46]. URF13 is a CMS protein in the maize CMS-T system, and it has an independent toxicity mechanism that causes male sterility in maize. Furthermore, when URF13 binds to specific ligands, it forms pores in the inner mitochondrial membrane, leading to loss of mitochondrial membrane potential and cell death, a further toxic mechanism of URF13 [3, 20].

The abortion mechanism of Ogura-CMS is generally believed to be related to cytotoxicity. ORF138 has been confirmed to be the gene responsible for pollen abortion in Ogura-CMS [10, 43]. The intact N-terminus of ORF138 can inhibit the growth of Escherichia coli. Genetic transformation experiments in *Arabidopsis thaliana* and rapeseed have demonstrated that the N-terminal toxic region and transmembrane structure of ORF138 are essential for causing male sterility in plants [5, 43]. However, the specific mechanism of pollen abortion in Ogura-CMS has not yet been determined. Mitochondria-targeted expression of the nuclear-encoded *orf138* in yeast and onion epidermal cells revealed altered mitochondrial cytological appearance in both cells. Mitochondrial morphological changes were also observed in *orf138* transgenic *Arabidopsis* pollen [6]. Expression of orf138 in *Arabidopsis* using a mitochondrial targeting vector resulted in male sterility, whereas nuclear expression resulted in normal fertility [6], suggesting that ORF138 controls male sterility in Ogura-CMS via mitochondria. The mitochondrial content of tapetal cells and microspore cells in pollen grains is 40 times and 20 times that of somatic cells, respectively. Mitochondrial abnormalities have a more serious impact on pollen development [14, 30]. By inhibiting pyruvate dehydrogenase activity in sugar beet anther mitochondria, leading to mitochondrial dysfunction, researchers successfully obtained a microspore abortion phenotype similar to that seen in sugar beet CMS plants [45]. Current research suggests that the cytotoxicity model essentially involves CMS proteins causing mitochondrial dysfunction in pollen sporophytes or gametophytes, leading to pollen abortion [2, 22]. However, experimental evidence remains lacking to explain how CMS proteins exert their toxicity and damage mitochondria.

3-hydroxypropionate is a toxic intermediate produced during the mitochondrial oxidation of propionate [48]. The MICOS complex regulates the contact between the inner and outer mitochondrial membranes and the formation of mitochondrial cristae [15, 37]. Previous studies in *Caenorhabditis elegans* have shown that 3-hydroxypropionate specifically binds to the Mic60/IMMT-1 subunit of the MICOS complex in mitochondria and inhibits Mic60/IMMT-1-mediated cristae formation, leading to mitochondrial damage [48]. ORF138 is located in the inner mitochondrial membrane of CMS plants and functions independently, may form pores in the inner mitochondrial membrane, which further causes male sterility [5, 7]. This study performed transcriptome and metabolome sequencing on the anthers of ZJCMS1A and ZJCMS1B at different developmental stages. Comprehensive analysis revealed a close relationship between 3-hydroxypropionic acid and Ogura-type CMS. Metabolomic data showed that the content of 3-hydroxypropionic acid in the anthers of ZJCMS1A was higher than that in ZJCMS1B at different stages of pollen abortion, and the content of 3-hydroxypropionic acid in the flower buds of ZJCMS2A was also higher than that in ZJCMS2B (Fig. 5E and F). The results of the substance content determination showed that the content of 3-hydroxypropionic acid in the tissues of ZJCMS1A was higher than that of the same tissues of ZJCMS1B, with the content in ZJCMS1A flower buds being 8.1% higher than in ZJCMS1B (Fig. 6A). These results indicate a close relationship between the increase in 3-hydroxypropionic acid content and pollen abortion in Ogura-type CMS lines. We attempted to treat Brassica napus protoplasts with 3-hydroxypropionic acid and detect changes in mitochondrial membrane potential using JC-1 staining, but the positive control experiment was consistently unsuccessful. Spraying Brassica napus plants at the initial flowering stage with 50 mM 3-hydroxypropionic acid showed a decrease in starch content in pollen grains after 6 days of treatment, with far more conspicuous results after 9 days (Fig. 6B). Similarly, at 20 mM, starch accumulation in the pollen grains was also observed to be affected after 7 days of treatment (S Fig. 3). This may be attributed to the abnormal accumulation of 3-hydroxypropionic acid, which damages the mitochondrial inner membranes within the pollen cells, thereby affecting starch accumulation in the pollen grains. Furthermore, 3-hydroxypropionic acid is weakly acidic, and its excessive accumulation may alter the intracellular pH of pollen cells, thereby affecting the starch synthesis process. In this study, whole rapeseed plants were subjected to spraying with 20 mM and 50 mM 3‑hydroxypropionic acid. To eliminate potential interference, no surfactants or other additives were included in the treatment solution. Additionally, we conducted trials using 3-hydroxypropionic acid solutions at concentrations of 100 mM, whereas at 100 mM, the whole plant turned yellow, and normal growth was inhibited. This study did not quantify the efficiency with which the rapeseed plants absorbed the 3-hydroxypropionic acid. Furthermore, given the scarcity of precedents for treating plants with 3-hydroxypropionic acid, it is possible that the sprayed solution evaporated rapidly. In summary, numerous uncertainties existed,ideally, the 3-hydroxypropionic acid content within the plants would have been monitored in real-time—a feat that was, however, clearly unfeasible. Ultimately, we opted for a strategy of continuous treatment and observation. Following a 9-day treatment period and a 3-day recovery phase, we found that the application of 3-hydroxypropionic acid influenced starch accumulation within the rapeseed pollen, albeit in a manner that could not be quantitatively measured. In future studies, we can modulate the core functional genes of the 3-Hydroxypropionyl-CoA metabolic pathway (Fig. 5D) to comprehensively unravel the mysteries underlying the relationship between 3-hydroxypropionic acid and CMS.

Another question is why the CMS gene only affects anther fertility, even though it can be expressed in other tissues [2]. Recent research in rice indicates that the CMS gene WA352 is constitutively expressed in CMS-WA, but it is degraded through ubiquitination in other tissues. However, WA352 can specifically accumulate in anthers, leading to male sterility [46]. In the Ogura-type CMS line, *orf138* is also constitutively expressed, but no damage to trophic organs has been found [8, 11]. This study found that the content of 3-hydroxypropionic acid in both ZJCMS1A and ZJCMS1B flower buds exceeded 10 μg/g, while it was below 5 μg/g in stems and leaves. Furthermore, the content of 3-hydroxypropionic acid in ZJCMS1A flower buds was 8.1% higher than that in ZJCMS1B flower buds. These results suggest a close relationship between the content of 3-hydroxypropionic acid and the function of *orf138*, and that pollen abortion in ZJCMS1A may be related to its high 3-hydroxypropionic acid content. This has provided a theoretical basis for the sterility mechanism of Ogura CMS in rapeseed and for hybrid seed production. In this study, the 3-hydroxypropionic acid content measured was from the flower buds of ZJCMS1A and ZJCMS1B, rather than that from the anthers. This approach numerically reduced the fold difference in 3-hydroxypropionic acid levels observed between ZJCMS1A and ZJCMS1B, given that anthers constitute only a small proportion of the flower bud, and the disparity in 3-hydroxypropionic acid is expected to be manifested primarily within the anthers themselves. Nevertheless, a significant difference in 3-hydroxypropionic acid content was still observed between the flower buds of ZJCMS1A and ZJCMS1B.

Transcriptome sequencing analysis of the anthers in Ogura-type CMS lines suggests that abnormal lipid metabolism may be a key factor affecting the male fertility of Ogura-type CMS lines [10]. This study performed transcriptome and metabolome sequencing on anthers of Ogura-type CMS lines at early, middle, and late abortion stages, and combined the results to find that the propanoate metabolic pathway and the fatty acid biosynthesis pathway had the greatest impact on pollen abortion in Ogura-type CMS lines. Abnormal fatty acid biosynthesis pathways can also lead to abnormal lipid metabolism. However, this study found that the abnormal accumulation of 3-hydroxypropionic acid in the propanoate metabolic pathway in ZJCMS1A/ZJCMS2A was more strongly correlated with the exertion of function of *orf138*. The results of the determination of 3-hydroxypropionic acid content in different tissues of ZJCMS1A and ZJCMS1B, as well as the observations of pollen from rapeseed treated with 3-hydroxypropionic acid, also demonstrate that the propionic acid metabolic pathway may plays an important role in the Ogura-type CMS process.

## Materials and methods

### Plant materials and growth conditions

The rapeseed (*Brassica napus*) cytoplasmic male sterile line ZJCMS1A possesses Ogura-type sterile cytoplasm, and its nucleus is derived from the cultivar *Zheyou51*. *Zheyou51* is the maintainer line of ZJCMS1A and is thus named ZJCMS1B. Similarly, the ZJCMS2A line possesses Ogura-type sterile cytoplasm, and its nucleus is derived from the cultivar *Zhongshuang 11*. The maintainer line ZJCMS2B is *Zhongshuang 11*. Rapeseed plants were planted and managed in the foundation seed farm of Hangzhou, Zhejiang Province. ZJCMS1A, ZJCMS1B, ZJCMS2A, and ZJCMS2B were planted in the autumn of 2023. The Zhejiang Academy of Agricultural Sciences provided all seeds. ZJCMS1A and ZJCMS1B used in the validation experiment were grown in a mixture of natural soil and nutrient soil (1:1). The environmental conditions were maintained at 18 °C in the dark for 8 h and 22 °C in the light for 16 h, in a cyclic manner.

### Pollen grain microscopic observation

Samples were collected from ZJCMS1A and ZJCMS1B during the flowering phase. Flower buds of 2 mm, 3 mm, and 4 mm in length were measured using a vernier caliper. The anthers were removed and quickly fixed in 2.5% glutaraldehyde fixative for 2–4 h. After rinsing three times with 0.1 M phosphate buffer (pH 7.0), the samples were fixed in phosphate buffer (PB) containing 1% osmium sulfate for 1–3 h. After rinsing, anther samples were dehydrated in a series of increasing alcohol concentrations (50%, 70%, 80%, 90%, 95%, 100%, and 100%) for 15 min each. Following dehydration, the samples were infiltrated in a 1:1 solution of 100% ethanol and isoamyl acetate for 30 min, followed by infiltration in pure isoamyl acetate overnight. The samples were dried using a critical point dryer and coated with metal powder using a high-vacuum ion sputtering apparatus. Observation and imaging were performed using scanning electron microscope (Hitachi, Regulus 8100).

### Transcriptome and metabolome sequencing

During the flowering period of ZJCMS1A and ZJCMS1B, bud length was measured using a vernier caliper. Pollen abortion in Ogura CMS plants generally occurs from the tetrad stage to the uninucleate microspore stage [10]. The samples were divided into three groups of less than 2 mm, 2–3 mm, and 3–4 mm. The anthers were removed from each bud and placed in cryovials immersed in liquid nitrogen. ZJCMS1A samples were designated A2, A3, and A4 according to their bud length ranges, while the corresponding ZJCMS1B samples were designated B2, B3, and B4. During sampling, the cryovials were fixed with the opening facing upward. After sufficient samples were obtained, they were temporarily kept in liquid nitrogen and quickly transferred to an ultralow-temperature freezer (−80 °C), and then sent to the company for sequencing.

RNA was extracted from samples for transcriptome sequencing, and RNA integrity was assessed using an Agilent 2100 bioanalyzer. Three biological replicates were established. Total RNA was enriched for mRNA using Oligo (dT) magnetic beads. After fragmentation, first-strand and second-strand cDNA synthesis were performed. Library construction was completed after A-tailing, adapter ligation, amplification, and purification. Initial quantification was performed using a Qubit 2.0 Fluorometer. The library was diluted to 1.5 ng/μl, and the insert size was measured using an Agilent 2100 bioanalyzer. The effective concentration of the library was determined by quantitative real-time PCR (qRT-PCR) to ensure library quality. Once the library passed the assay, Illumina sequencing was performed. Raw sequence data were obtained, quality control was performed, and clean reads were filtered. Clean reads were aligned to the reference genome, version ZS11.V0 [34], using HISAT2 software. The featureCounts tool in subread software was used to quantify reads for different genes [23]. Statistical analysis was performed based on the quantitative gene expression data to screen genes with significant differences in expression levels between different samples. The screening criteria for differentially expressed genes are |log2(FoldChange)|> = 1 and padj < = 0.05.

Samples obtained from the wild field were freeze-dried and ground into powder. 50 mg of each sample powder was weighed and added to 1200 μL of a 70% methanol–water internal standard extract (−20 °C). The mixture was vortexed six times at 30-min intervals prior to centrifugation. The supernatant was aspirated and filtered through a 0.22 μm pore size microfilter and stored in a vial. Ultra Performance Liquid Chromatography (UPLC) (ExionLC™AD) and Tandem mass spectrometry (MS/MS) analysis were performed. Mass spectrometry data were processed using Analyst 1.6.3 software, and qualitative and quantitative analysis of metabolites was performed using the Metware (Wuhan MetWare Biotechnology Co., Ltd.) Metabolomics Database, and the analysis was performed using the OPLSR. Anal function in the MetaboAnalystR package in R software. Variable Importance in Projection (VIP), derived from an OPLS-DA model (biological replicates ≥ 3), was used to identify metabolites that differed between tissues. In metabolome analysis, a VIP > 1 is generally considered significant.

### Comparative grouping and analysis

Samples were grouped for comparative analysis. A2_vs_B2, A3_vs_B3, and A4_vs_B4 were used to identify different genes and metabolites between ZJCMS1A and ZJCMS1B at different anther developmental period. Venn analysis was performed on the differentially expressed genes and metabolites in the A2_vs_B2, A3_vs_B3, and A4_vs_B4 comparisons to identify genes and metabolites that specific changed between ZJCMS1A and ZJCMS1B at different period. A2_vs_A3, A3_vs_A4, and A2_vs_A4 were used to identify genes and metabolites that changed during ZJCMS1A development. B2_vs_B3, B3_vs_B4, and B2_vs_B4 served as controls to reflect changes in genes and metabolites during normal pollen development. Venn analysis was performed on the six comparison groups of A2_vs_A3, A3_vs_A4 and A2_vs_A4, as well as B2_vs_B3, B3_vs_B4 and B2_vs_B4 to identify the different genes and metabolites that changed specifically in ZJCMS1A or ZJCMS1B between different developmental stages.

### Multi-omics integration KEGG enrichment analysis

The KEGG pathways in the Kyoto Encyclopedia of Genes and Genomes (KEGG) database provide functional networks of biological systems constructed by genes and compounds. KEGG enrichment analysis can be used to integrate functionally related genes and metabolites into common KEGG pathways, which is commonly used in transcriptome and metabolome analysis. Multi-omics integration KEGG pathway analysis was performed on differentially expressed genes and metabolites in different comparison groups. Bubble plots were created using the R package ggplot2_3.3.6 to display the pathway intersections of metabolome and transcriptome enrichment results.

### KGML analysis

KEGG Markup Language (KGML) analysis can visualize genes and metabolic pathways in KEGG pathways, and show the connections between different KEGG pathways. KGML files were obtained from the KEGG pathway database and analyzed using the R package igraph_1.3.4 to generate network graphs.

### Total RNA isolation and quantitative Real-time PCR

Total RNA was extracted from rapeseed flower buds at various developmental stages using TRIzol reagent according to the manufacturer's protocol. cDNA was synthesized using the PrimeScript™ II 1 st Strand cDNA Synthesis Kit (Takara). Reaction systems were prepared using TB Green® Premix Ex Taq™ II (Takara) and performed on a real-time PCR instrument (ABI 7500). The internal control gene was *BnACT7* [27], and each reaction included three biological replicates. Relative gene expression levels were analyzed using the 2^−ΔΔCT^ method [25].

### Determination of 3-hydroxypropionic acid content

Weigh 3-hydroxypropionic acid (CAS: 503–66-2), dissolve it in methanol, and dilute it to eight concentrations ranging from 0.05 μg/mL to 100 μg/mL. Take 50 μL of each concentration solution and react it with 50 μL of pyridine and 250 μL of BSTFA solution for 1 h (70 ℃), respectively. After filtration through an organic filter membrane, take 1 μL of the secondary filtrate and analyze it using a gas chromatography-tandem mass spectrometry (GC–MS) analyzer (8890-7000D, Agilent). Plot a standard curve. Pulverize the flower buds, stems, and leaves of the test samples ZJCMS1A and ZJCMS1B, respectively. Weigh 0.1 g of each sample, add 1.0 mL of methanol, and mix well. Then, sonicate at room temperature for 30 min. The solution was then centrifuged for 5 min (5 °C, 7000 rpm) using a refrigerated centrifuge. 50 μL of the supernatant was added to 50 μL of pyridine and 250 μL of BSTFA solution and reacted for 1 h (70 °C). After filtration through an organic filter membrane, 1 μL of the secondary filtrate was analyzed using an analyzer (8890-7000D, Agilent). Statistical analysis was performed using GraphPad Prism 9.5.1 software.

### Treatment with 3-hydroxypropionic acid and observation of pollen viability

The working solution with a concentration of approximately 50 mM was prepared using 3-hydroxypropionic acid (CAS: 503–66-2, 28–32% in water) as the stock solution. Rapeseed plants in the early flowering stage were selected. The experimental group was sprayed with the working solution on the whole rapeseed plant at 4 PM daily for 12 consecutive days, while the control group was sprayed with pure water. The spray solution was in the form of a mist. Following the spray treatment, all rapeseed plants grew normally, and no visible damage was observed in any of their tissues. Flower buds were collected at 9 AM the day after each spraying and preserved in a 75% ethanol solution. Pollen grains were stained with I-KI Solution, and pollen grain viability was observed and images were acquired using a microscope (Leica, M165FC).

## Supplementary Information


Supplementary Material 1.
Supplementary Material 2.


## Data Availability

All data relevant to the results of this study are available in the paper and its supplementary materials. The datasets generated during the current study are available in the NCBI Sequence Read Archive (SRA) repository, under accession number: PRJNA1456214 (https://www.ncbi.nlm.nih.gov/bioproject/PRJNA1456214).

## Abbreviations

3-HP: 3-Hydroxypropionic acid
CMS: Cytoplasmic Male Sterility
GMS: Genic Male Sterility
ORF: Open Reading Frame
URF: Unidentified Reading Frame
CMS-Ogu: Ogura Cytoplasmic Male Sterility
mtETC: Mitochondrial electron transport chain
PCD: Programmed cell death
*Rf*: Fertility Restore gene
RMS: Retrograde-regulated male sterility
ATP: Adenosine Triphosphate
DEGs: Differentially Expressed Genes
KEGG: Kyoto Encyclopedia of Genes and Genomes
KGML: KEGG Markup Language
JC-1: 5,5',6,6'-Tetrachloro-1,1',3,3'-tetraethylbenzimidazolylcarbocyanine iodide
ROS: Reactive Oxygen Species
MICOS: Mitochondrial Contact Site and Cristae Organizing System
qRT‑PCR: Quantitative Real-Time Reverse Transcription Polymerase Chain Reaction
