# Supplementary material for: 3-Hydroxypropanoic acid contributing to pollen abortion in ogura cytoplasmic male sterility of Brassica napus
Source: BMC Plant Biol. 2026 Jun 18;26:1475. doi: 10.1186/s12870-026-09278-z (PMC13523307; doi:10.1186/s12870-026-09278-z)
Supplement: Supplementary file 1 [file 12870_2026_9278_MOESM1_ESM.docx]

**3-Hydroxypropanoic acid Mediates Pollen Abortion in Ogura Cytoplasmic Male Sterility of *Brassica napus***

Tanliu Wang, Jianghua Shi, Ying Fu, Huasheng Yu, Yaofeng Zhang*, Xiyuan Ni*

Key Laboratory of Digital Upland Crops of Zhejiang Province, Institute of Crop and Nuclear Technology Utilization, Zhejiang Academy of Agricultural Sciences, Hangzhou, China

* Corresponding author

Supplementary Figure 1: Multi-omics Integration KEGG Analysis.

A: Multi-omics Integration KEGG Analysis of differentially expressed genes and metabolites that uniquely exist in the A2_vs_B2 comparison. B: Multi-omics Integration KEGG Analysis of differentially expressed genes and metabolites that commonly exist in the A2_vs_B2, A3_vs_B3, and A4_vs_B4 comparison. C: Multi-omics Integration KEGG Analysis of differentially expressed genes and metabolites that uniquely exist in the A3_vs_A2, A4_vs_A3, and A4_vs_A2 comparison. D: Multi-omics Integration KEGG Analysis of differentially expressed genes and metabolites that uniquely exist in the B3_vs_B2, B4_vs_B3, and B4_vs_B2 comparison.

Supplementary Figure 2. qRT-PCR identification of the expression levels of genes related to 3-hydroxy-propionyl-CoA regulation. Asterisks indicate significant differences, and ns indicates no significant difference.

Supplementary Figure 3. Microscopic observation of pollen grains. Rapeseed plants were sprayed with 3-hydroxypropionic acid at 20 mM and 50 mM. Pollen grains were observed using iodine-potassium iodide staining on days 4 and 7 after treatment, as well as on 3 days post-treatment. Darker color indicates higher starch content and better pollen viability. Lighter black indicates low starch content and abnormal pollen development. Darker color indicates higher starch content and better pollen grain viability. Lighter color indicates lower starch content and abnormal pollen grain development.
